# Differences in the gut microbiota between Gurkhas and soldiers of British origin

**DOI:** 10.1371/journal.pone.0292645

**Published:** 2023-12-19

**Authors:** Thomas D. Troth, Ross S. McInnes, Steven J. Dunn, Jeremy Mirza, Annalise H. Whittaker, Sarah A. Goodchild, Nicholas J. Loman, Sarah V. Harding, Willem van Schaik

**Affiliations:** 1 Institute of Microbiology and Infection, University of Birmingham, Birmingham, United Kingdom; 2 CBR Division, Defence and Science Technology Laboratory, Salisbury, Wiltshire, United Kingdom; 3 School of Respiratory Sciences, University of Leicester, Leicester, United Kingdom; Washington State University - Spokane, UNITED STATES

## Abstract

Previous work indicated that the incidence of travellers’ diarrhoea (TD) is higher in soldiers of British origin, when compared to soldiers of Nepalese descent (Gurkhas). We hypothesise that the composition of the gut microbiota may be a contributing factor in the risk of developing TD in soldiers of British origin. This study aimed to characterise the gut microbial composition of Gurkha and non-Gurkha soldiers of the British Army. Recruitment of 38 soldiers (n = 22 Gurkhas, n = 16 non-Gurkhas) and subsequent stool collection, enabled shotgun metagenomic sequencing-based analysis of the gut microbiota. The microbiota of Gurkhas had significantly (P < 0.05) lower diversity, for both Shannon and Simpson diversity indices, using species level markers than the gut microbiota of non-Gurkha soldiers. Non-metric Multidimensional Scaling (NMDS) of the Bray-Curtis distance matrix revealed a significant difference in the composition of the gut microbiota between Gurkhas and non-Gurkha soldiers, at both the species level (P = 0.0178) and the genus level (P = 0.0483). We found three genera and eight species that were significantly enriched in the non-Gurkha group and one genus (*Haemophilus*) and one species (*Haemophilus parainfluenzae*) which were enriched in the Gurkha group. The difference in the microbiota composition between Gurkha soldiers and soldiers of British origin may contribute to higher colonization resistance against diarrhoeal pathogens in the former group. Our findings may enable further studies into interventions that modulate the gut microbiota of soldiers to prevent TD during deployment.

## Introduction

Travellers’ diarrhoea (TD), a gastroenteritis primarily mediated by bacteria, is a significant issue for people visiting low and middle income countries [1], with research showing that multiple species and strains of bacteria can transiently or chronically colonise the lower intestinal tract [2]. Although typically short in its duration (<2 weeks) and largely self-limiting, the impact of travellers’ diarrhoea can be substantial, and patients can develop post-infectious sequelae that lead to chronic illness (e.g. irritable bowel syndrome) [3, 4].

The detrimental effects of TD on deploying British forces is well documented, as far back as the Crusades [5]. Diarrhoeal disease remains a significant cause of Disease, Non-Battle Injury for deployed troops. Self-reported travellers’ diarrhoeal rates were approximately 40% during two separate Operation HERRICK (Afghanistan: 2002–2014) tours [6]. A large proportion of soldiers suffering from travellers’ diarrhoea reported a reduction in the ability to work (~50%), a quarter were incapacitated, and a small number required hospitalisation [7]. The average “days off duty” with TD was 2.8, with an average of 4 days of underperforming per episode. This resulted in an (extrapolated) aggregated 68,918 man-days lost to underperformance over 2 x 6 month deployments to Afghanistan [6].

Amongst members of the British Army, travellers’ diarrhoea primarily affects soldiers born in the UK. However, in addition to British nationals, the British Army also recruits from other countries, including British Gurkha soldiers originating from South Asia (primarily Nepal). Gurkha soldiers have demonstrated a resistance to bacterial gastroenteritis during overseas deployment, with the incidence of diarrhoeal episodes reduced when compared to non-Gurkha soldiers (11% and 40–60% respectively) [6]. The mechanisms underpinning this reduction in incidence are unknown, however understanding this may provide important therapeutic and prophylactic treatment options that could significantly mitigate the impact of travellers’ diarrhoea in a military context.

Research has shown that differences in the gut microbiota are associated with the incidence of diarrhoeal episodes [8], and the composition of the gut microbiota can vary greatly depending on a variety of host factors, including diet, lifestyle and geographical origin [9–11]. A key theme in gut microbiota research is assessing the impact that an individual’s microbiota can have on the likelihood of developing disease, with many studies attempting to understand how these factors are linked [12–15].

This study aimed to characterise the composition of soldiers’ gut microbiota, and to assess whether there were any differences in composition between Gurkha and non-Gurkha soldiers.

## Methods

### Participant recruitment

This study was reviewed and received a favourable opinion from the Ministry of Defence Research and Ethics Committee (MODREC) reference 2019/MODREC/21. All participants were briefed on the study’s objectives and were provided with patient information leaflets before signing consent forms. Samples were provided in private and returned to the study team. Each individual was randomly allocated a study number from 1–38 and anonymised data were further processed during the analysis. Exclusion criteria were any chronic underlying medical condition, acute gastrointestinal upset (diarrhoea or vomiting), recent (<3 months) travel abroad, SARS-CoV-2 infection, or antibiotic use.

A total of 38 soldiers were recruited from 3 barracks across England, which were categorised according to their reported ethnicity and country of birth. A total of 22 non-UK born Gurkhas were recruited. 12 UK born non-Gurkhas were recruited, and 4 soldiers were born in non-UK countries, but were not Gurkhas. The group defined as ‘Gurkha’ comprises soldiers that are first generation Gurkhas born outside of the UK, and ‘non-Gurkha’ are all those who do not meet this criterion. Thirty-seven of the participants were male, the one female was part of the ‘non-UK, non-Gurkha’ group.

### Sample collection

Volunteers were provided with the OMNIgene Gut OMR-200 collection kit (DNA Genotek, Ontario, Canada) for self-directed collection. Participants were asked to collect a single sample of faecal material, which was subsequently placed inside a collection tube containing a stabilisation buffer and a metal ball-bearing that rapidly homogenised the sample. Homogenised and stabilised samples were cryopreserved at -80˚C for long-term storage.

### DNA extraction

Faecal samples were thawed, and 200 mg of material was processed using the FastDNA Spin Kit for Soil (MP Biomedicals, California, USA). Samples were aliquoted into tubes containing ceramic beads of varying size, processed using a FastPrep-24 5G bead beating machine (MP Biomedicals, California, USA), and subsequently treated following the protocol of the FastDNA Spin Kit for Soil.

In addition to the volunteer samples, the ZymoBiomics Fecal Reference with TruMatrix Technology (Zymo Research, California, USA) was included as a positive control. This was included to control for the consistency and composition of faecal material, and contained a defined bacterial composition. A DNA extraction was also performed on 100 μL of the stabilisation buffer from an unused OMNIGene Gut OMR-200 kit as a negative control, allowing for the detection of contamination from the sampling, extraction kits or procedures. Samples were processed in two batches. A single positive and negative control was included per batch.

### DNA sequencing

In addition to the controls prepared during the DNA extraction, a no-template negative control (dH_2_O), and a PhiX positive control were included in the library preparation and sequencing processes. Samples and controls were prepared using the standard Illumina DNA Prep protocol (Illumina, California, USA). Libraries were quantified using a Qubit 4 Fluorometer (Thermofisher Scientific, Massachusetts, USA) and normalised to 100 ng in 30 μL by diluting with nuclease-free water (negative controls contained no detectable DNA and were not diluted). The concentration used was the lowest recommended for this protocol to reduce the risk of inhibiting tagmentation.

Following preparation, the pooled libraries were run on a 2200 Tapestation (Agilent) with a D1000 screentape to obtain an average library size of 571 base pairs (bp) at a concentration of 12 nM. This concentration was diluted to 4 nM as per the recommendations for the library and the final concentration was confirmed using the NEBNext Library Quant Kit for Illumina (New England Biolabs, Massachusetts, USA).

The prepared libraries were initially run on an Illumina MiSeq to assess their quality and equimolarity. The final 4 nM library was denatured and diluted as per Protocol A of the MiSeq System guide. A final concentration of 15 pM was loaded onto the cartridge to optimise cluster density and was run using a 500-cycle kit and flow cell with version 2 reagents (Illumina, California, USA).

Following this run, a small number of indices were identified that required adjustment for equimolarity. Following this adjustment, the library was denatured and diluted using the HiSeq Guide (Protocol A: Standard Normalisation Method). The denatured library was diluted to a final concentration of 8 pM and loaded onto the HiSeq 2500 system. The HiSeq was run in rapid run mode with version 2 reagents of the Rapid SBS Kit and Rapid PE Cluster Kit for 500 cycles (Illumina, California, USA). HiSeq bcl files were converted to fastq files using bcl2fastq (V. 2.19.0).

### Sequence analysis

Reads were processed using fastp (V. 0.23.2; [16]) under default parameters to remove adapter content and perform quality-based trimming of reads. Read quality was assessed using FastQC (V. 0.11.5; https://www.bioinformatics.babraham.ac.uk/projects/fastqc/) and MultiQC (V. 1.6; [17]). Following quality trimming, the median number of bases per sample was 2.19 Gb, with the majority of samples at or close to equimolar ratio (S1 Fig). The mean read length was 246.1 bp (SD = 9.965). All negative controls yielded a total of 0 megabases. Prior to read upload, human reads were removed from all samples using bbmap (V. 38.06; [18]) and the hg19 human reference genome.

Microbial abundance was analysed using a marker-based method implemented in MetaPhlAn3 (V. 3.0.14; [19]), in conjunction with the CHOCOPhlAn marker database (v. 30). Further analysis and data visualisation was conducted using R (V. 4.1.2) and the gplots, ggplot2, ggfortify, ggrepel, ggpubr, showtext, dplyr, tidyverse, reshape2, and viridis packages.

### Statistical analysis

Statistical analyses were also performed in R using the adonis, diversity and metaMDS functions from the vegan package. An association analysis between taxa (species- and genus-level) and group (Gurkha and non-Gurkha) was performed using MaAsLin2 (V. 1.4.0; [20]).

## Results

### *Prevotella* and *Bacteroides* are highly abundant in the British soldier’s gut microbiota

**Fig 1:**
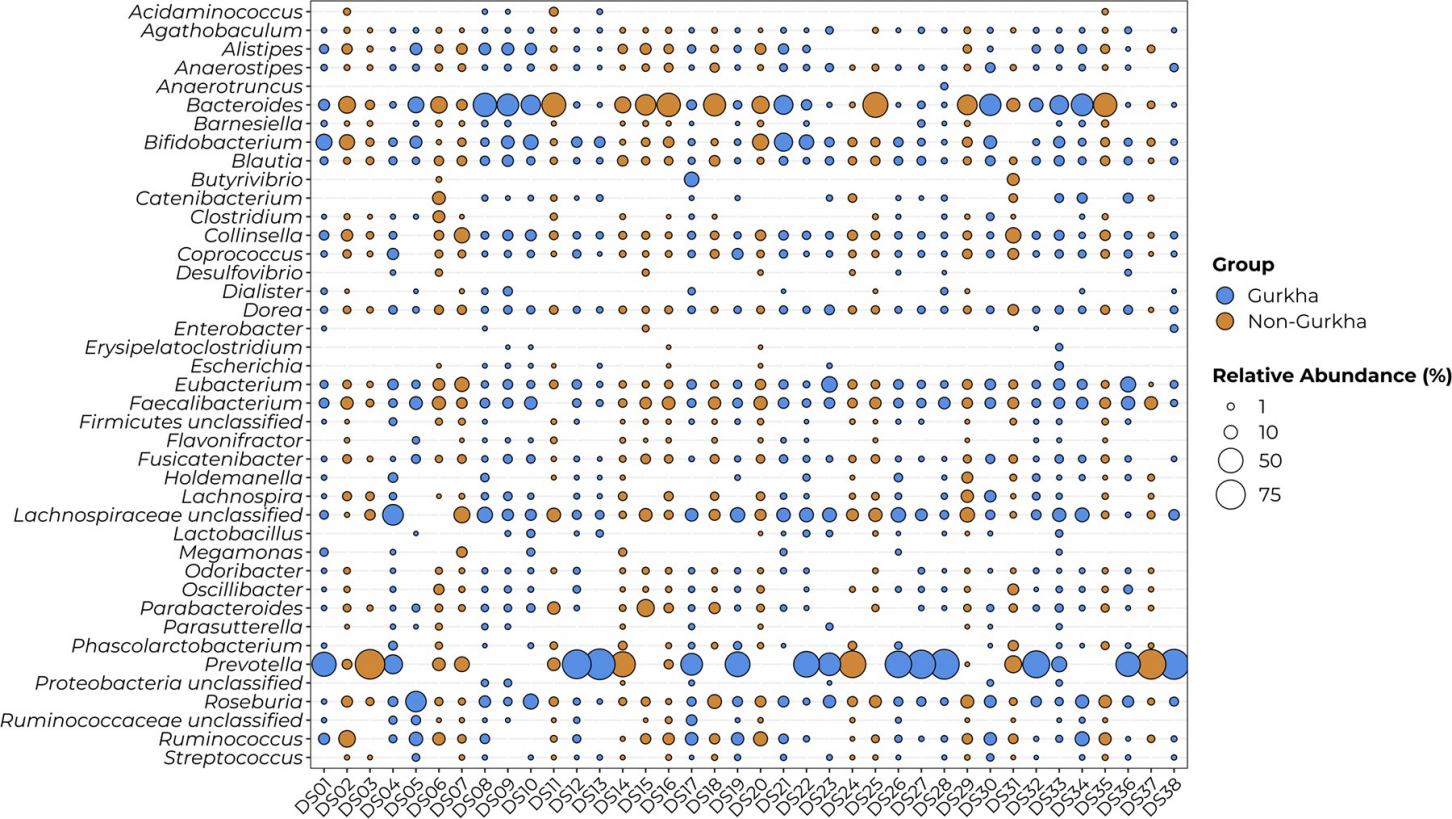
Genus level composition of British soldier’s gut microbiota. The composition of British soldier’s gut microbiome samples at the genus level. The size of the circle represents the relative abundance of the genus in a sample and the colour represents, Gurkha (blue) or Non-Gurkha (gold). Only genera that had a relative abundance greater than 1% in one or more samples were included.

The gut microbiota samples of British soldiers contained a total of 41 unique genera with a relative abundance greater than 1% (Fig 1) in at least one of the samples. The most widespread of these genera were *Bacteroides*, *Blautia*, *Dorea*, *Eubacterium* and *Roseburia* which were present in all samples. The genus *Anaerotruncus* was the least widespread and was found in only one sample. *Prevotella* was the most abundant genus in the faecal samples with a median relative abundance of 17% (Interquartile Range [IQR]: 0% to 59%). The genus *Bacteroides* was also highly abundant in the samples with a median relative abundance of 14% (IQR: 1% to 31%). All other genera composing the gut microbiota were found at median relative abundances less than 10% in all samples.

### Gut microbiota of Gurkha soldiers is less diverse than the gut microbiota of non-Gurkhas

**Fig 2:**
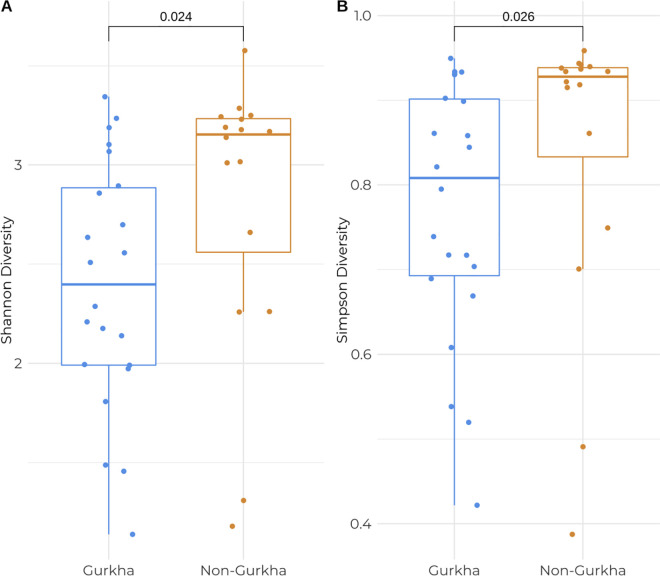
Diversity analysis of species-level markers. A. Shannon diversity. B. Simpson diversity in Gurkha and non-Gurkha soldiers using species-level markers. Statistical analysis was performed using the Wilcoxon rank-sum test.

Diversity in the composition of the gut microbiota in Gurkha and non-Gurkhas were quantified using Shannon and Simpson diversity indices. Using species-level markers, the gut microbiota of Gurkhas had a significantly lower diversity (P < 0.05, for both indices) than the gut microbiota of non-Gurkha soldiers (Fig 2). However, on the genus level there was no significant difference in diversity (P = 0.084 for Shannon diversity; P = 0.16 for Simpson diversity) between both groups (S2 Fig).

### The gut microbiota composition is significantly different between Gurkha soldiers and non-Gurkha soldiers

**Fig 3:**
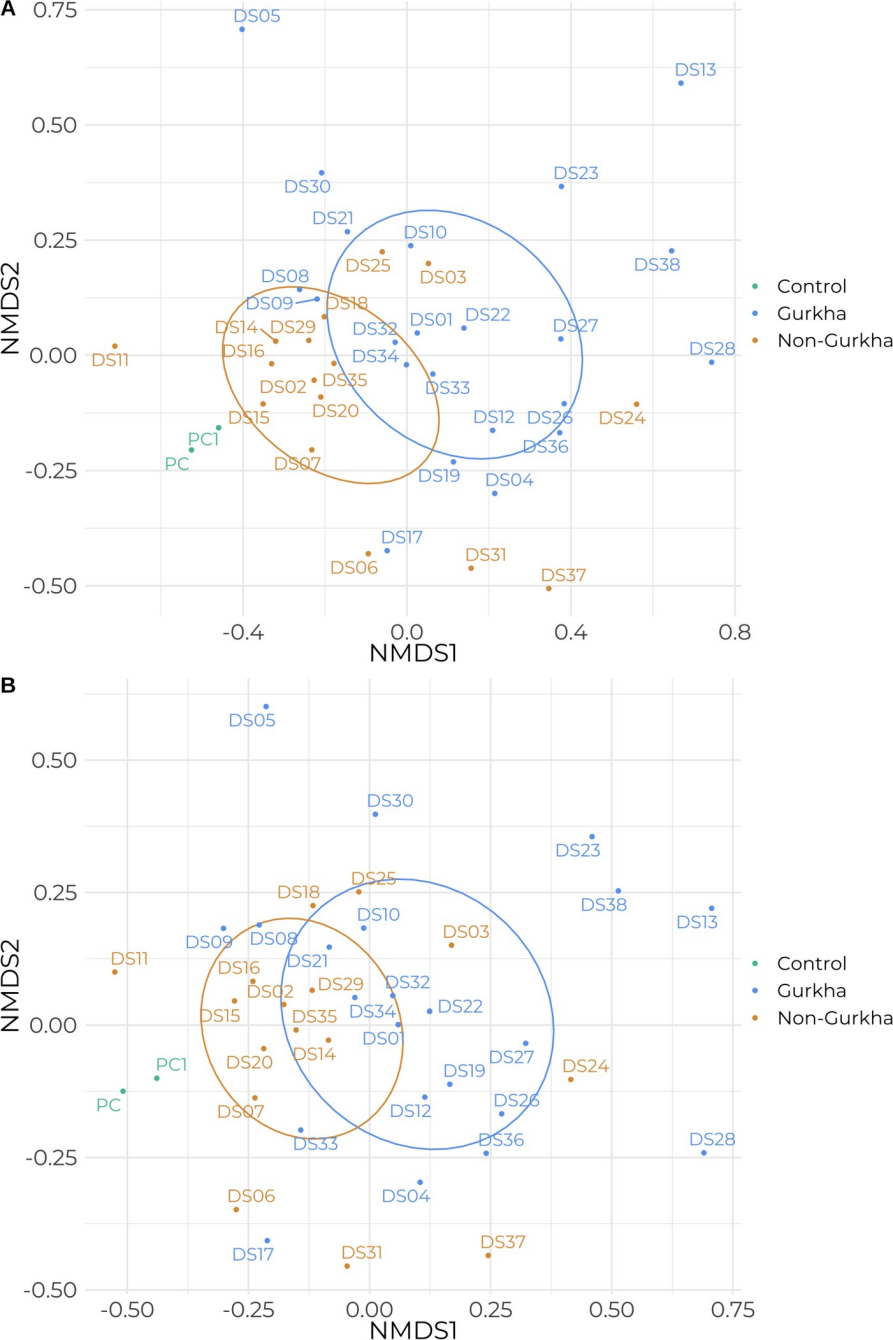
Microbiota composition analysis of Gurkhas and non-Gurkhas. Non-metric Multidimensional Scaling (NMDS) distribution of the Bray-Curtis distance matrix generated from species-level abundance (panel A) and genus-level abundance (panel B) is shown. Statistical testing of the differences in microbiota composition between the two groups (panel A, P = 0.0178; panel B, P = 0.0483) was performed by running 10,000 instances of a permutational multivariate analysis of variance (PERMANOVA). Stress: panel A: 0.19, panel B: 0.2. 50% confidence interval ellipses are indicated. The ZymoBiomics Fecal Reference with TruMatrix Technology (Zymo Research, California, USA) was included as a positive control.

To further asses the differences in the gut microbiota between both groups we used non-metric Multidimensional Scaling (NMDS) of the Bray-Curtis distance matrix generated from species-level and genus-level abundance (Fig 3). This revealed a significant difference in the composition of the gut microbiota between Gurkhas and non-Gurkha soldiers, at both the species level (P = 0.0178) and the genus level (P = 0.0483). Participant age and base of collection were non-significant factors at the species level (P = 0.1369, 0.2128) and the genus level (P = 0.1829, 0.2878).

To further characterise the differences in the composition of the gut microbiota, we used MaAsLin 2 [20] to identify taxa that were specifically enriched in either of the two groups. We found three genera and eight species that were significantly enriched in the non-Gurkha group and one genus (*Haemophilus*) and one species (*Haemophilus parainfluenzae*) which were enriched in the Gurkha group (Table 1).

**Table 1:** Summary of MaAsLin2-derived associations between taxa in the gut microbiota and the two groups of soldiers (Gurkha and non-Gurkha).

taxon (genus)	coefficient	standard error	FDR-adjusted P-value
*Asaccharobacter*	1.05	0.29	0.0466
*Parabacteroides*	0.69	0.21	0.0466
*Ruthenibacterium*	0.60	0.19	0.0466
*Haemophilus*	-0.62	0.19	0.0466
**taxon (species)**			
*Alistipes finegoldii*	1.20	0.24	0.0023
*Bacteroides faecis*	0.76	0.17	0.0048
*Firmicutes* bacterium CAG 83	0.81	0.20	0.0111
*Asaccharobacter celatus*	1.05	0.29	0.0283
*Bacteroides cellulosilyticus*	0.51	0.14	0.0283
*Parabacteroides distasonis*	0.84	0.24	0.0283
*Clostridium* sp CAG 58	0.44	0.13	0.0357
*Haemophilus parainfluenzae*	-0.79	0.24	0.0429
*Ruthenibacterium lactatiformans*	0.59	0.19	0.0455

The positive numbers under coefficient indicate taxa that are significantly (Benjamini-Hochberg adjusted P-value < 0.05) enriched in the non-Gurkha group, while negative numbers indicate taxa that are enriched in the Gurkha group.

## Discussion

An individual’s microbiota can vary dramatically on a person-to-person basis, with a wide array of influencing factors. Ethnicity has been identified as a factor in determining the composition of the gut microbiota, with populations of different ethnicities sharing the same geography having notable differences in their composition of the gut microbiota [21, 22]. Many factors, including differences in socioeconomic status, lifestyle, and environmental and dietary factors, may explain how ethnicity can impact the composition of the gut microbiota between different ethnic groups. We note that it is a limitation of our study that we did not collect data on different potential drivers of microbiome composition.

In this pilot study, we observed significant differences in the gut microbiota of Gurkha soldiers and non-Gurkha soldiers in the British Army. Gurkhas had a lower diversity of their microbiota and non-Gurkhas had significantly higher levels of several gut commensals from the phyla Bacteroidetes and Firmicutes. The only taxon that was found to be enriched in the Gurkha gut microbiota was *Haemophilus parainfluenzae*.

*H*. *parainfluenzae* is an opportunistic pathogen which is ubiquitously found in the human upper respiratory tract [23], but is also commonly detected in the human gut microbiota [24]. The role of *H*. *parainfluenzae* in the gut is poorly characterised. Specific strains of *H*. *parainfluenzae* have been associated with gut inflammation and inflammatory bowel disease [25], but its abundance is positively correlated with markers of nutritional and cardiometabolic health [24]. Further studies may be needed to better characterise the function of *H*. *parainfluenzae* in the gut microbiota.

On the basis of the data reported here, we cannot determine whether the differences in the gut microbiota between Gurkha and non-Gurkhas could be responsible for the differences in the risk of traveller’s diarrhoea between both groups. While a higher gut microbiota diversity is generally perceived as contributing to gut health [26], it is notable that in a study in 11 American soldiers the baseline microbiota diversity did not impact risk of diarrhoea upon deployment in Central America [27].

Whilst the number of samples used in this initial study is low (n = 38), it is possible to observe a split in the microbial composition of the microbiota of Gurkha versus non-Gurkha soldiers. To fully characterise the gut microbiota’s role in providing resistance against traveller’s diarrhoea in military personnel, observational studies of cohorts of soldiers during deployment abroad will need to be performed. In addition, to better understand the drivers of gut microbiota composition among ethnically diverse cohorts, additional information on diet needs to be collected to determine the potential contribution of dietary factors in shaping the gut microbiota.

## Supporting information

S1 Table Sample metadata and their associated biosample and read accession numbers. All data is deposited under Bioproject accession number PRJNA849795. Bases on which sample collection took place have been anonymised.
(XLSX)

S1 Fig Total number of bases of sequencing data generated per sample. A median of 2.19 gigabases was generated per sample. The negative controls did not produce any sequencing data. The Gurkha group is defined as first-generation Gurkha soldiers born outside of the UK. The ZymoBiomics Fecal Reference with TruMatrix Technology (Zymo Research, California, USA) was included as a positive control (PC and PC1).
(PDF)

S2 Fig Diversity analysis of genus-level markers. A. Shannon diversity. B. Simpson diversity in Gurkha and non-Gurkha soldiers using genus-level markers. Statistical analysis was performed using the Wilcoxon rank-sum test.
(PDF)
