# Supplementary figures and images for: Differences in the gut microbiota between Gurkhas and soldiers of British origin
Source: PLoS One. 2023 Dec 19;18(12):e0292645. doi: 10.1371/journal.pone.0292645 (PMC10729956; doi:10.1371/journal.pone.0292645)

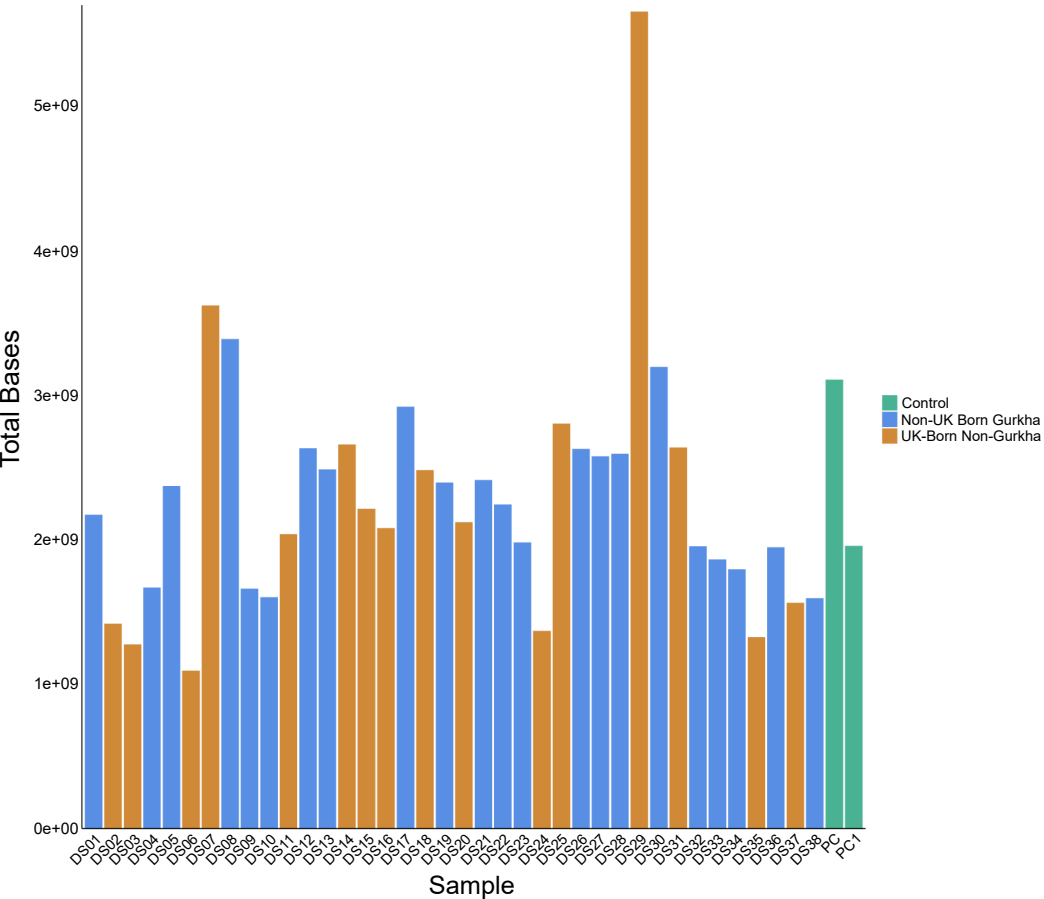

Supplement: S1 Fig — A median of 2.19 gigabases was generated per sample. The negative controls did not produce any sequencing data. The Gurkha group is defined as first-generation Gurkha soldiers born outside of the UK. The ZymoBiomics Fecal Reference with TruMatrix Technology (Zymo Research, California, USA) was included as a positive control (PC and PC1). (PDF) [file pone.0292645.s002.pdf]

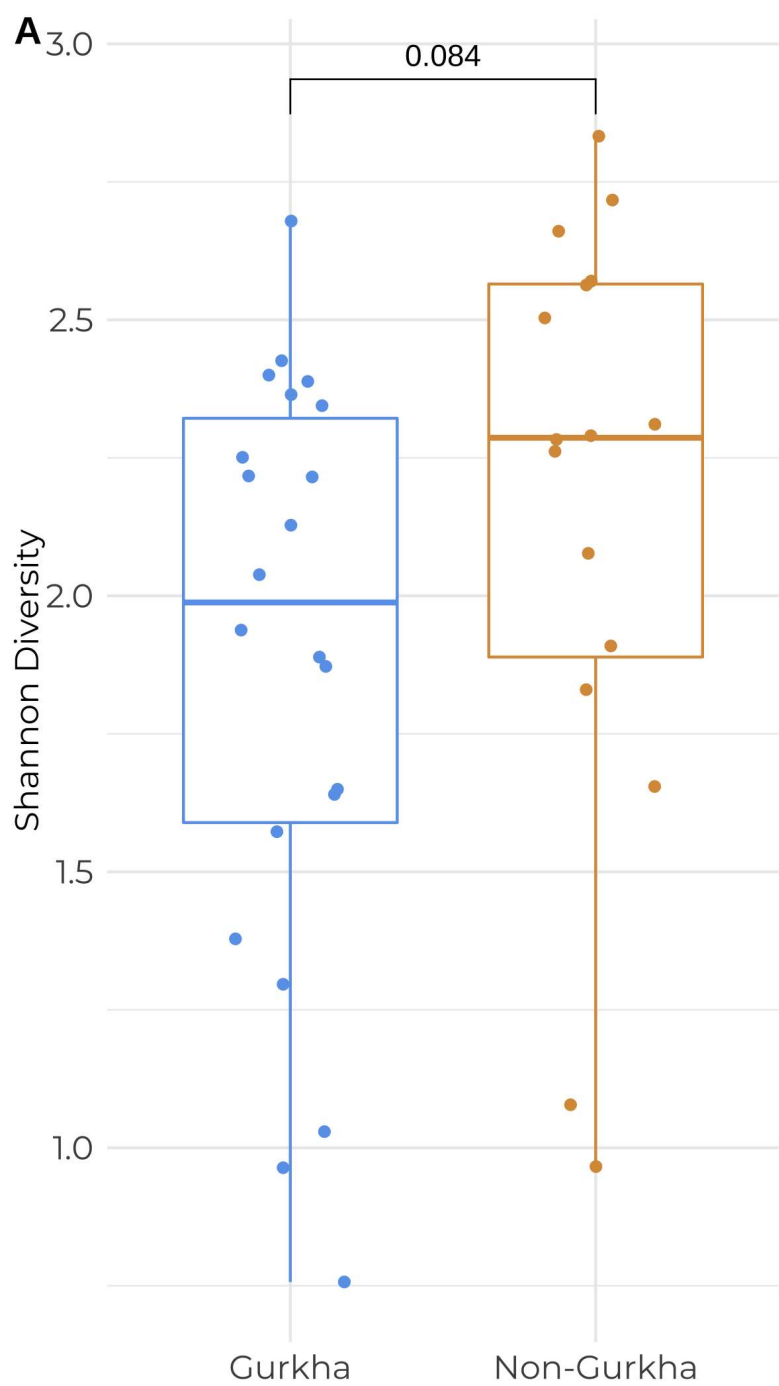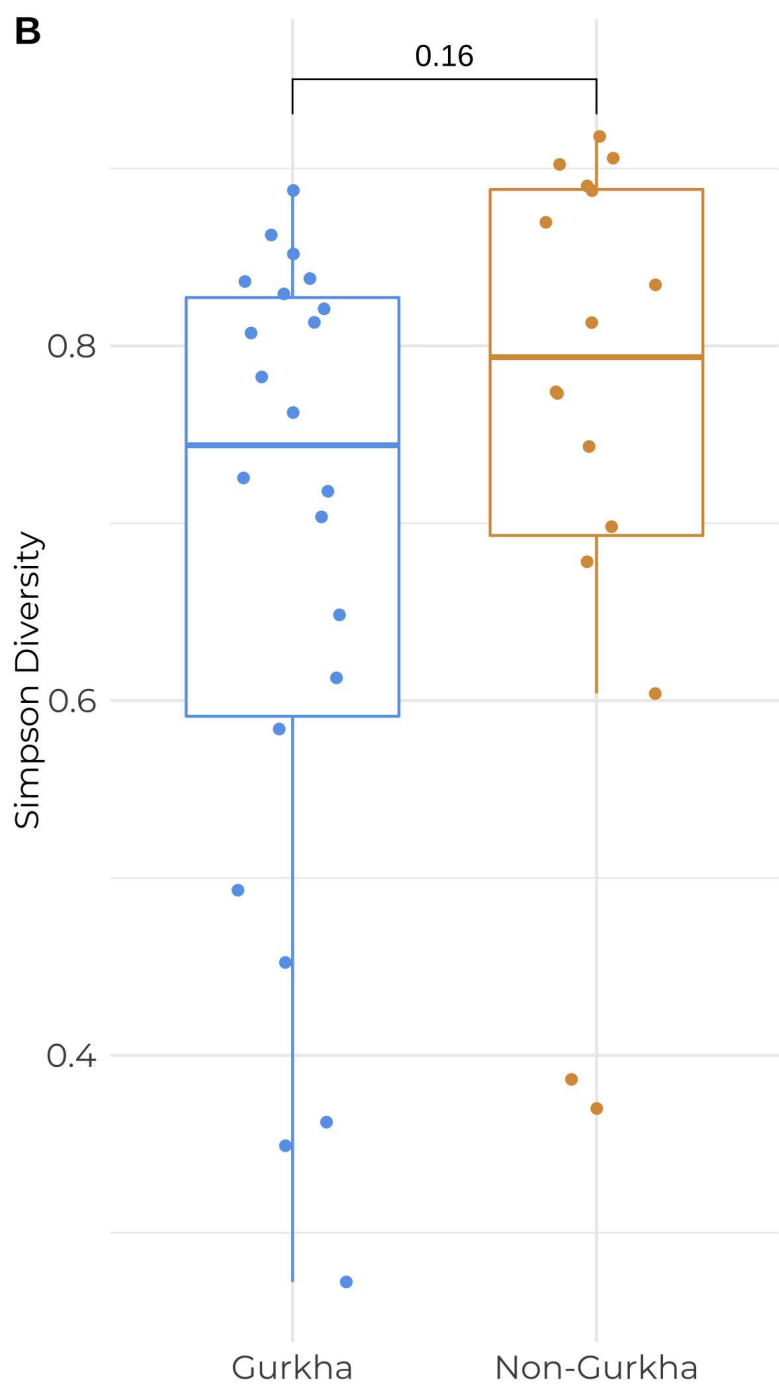

Supplement: S2 Fig — A. Shannon diversity. B. Simpson diversity in Gurkha and non-Gurkha soldiers using genus-level markers. Statistical analysis was performed using the Wilcoxon rank-sum test. (PDF) [file pone.0292645.s003.pdf]
